# Education—Past and Future

**Published:** 1902-05-31

**Authors:** 


### The Hospital

A Journal of

The Medical Sciences and Hospital Administration.

Vol. XXXII.—No. 818. Edited by Sir Henry Burdett, K.C.B., and Solomon C. Smith, M.D., M.R.C.P. [May 31, 1902.

### Education—Past and Future.

The large majority by which the Government carried the second reading of their Education Bill, and the willingness expressed by Mr. Chamberlain, in his recent speech at Birmingham, to preserve an open mind with regard to the details of its provisions, and to accept in Committee any amendments which would not sacrifice the general principles upon which the measure is based, are full of encouragement for those who desire to see the schools of the future rendered really conducive to the intellectual and practical advancement of the people. The time has therefore come at which it behoves all, who are themselves sufficiently educated to be able to comprehend the importance of the subject, to exert themselves strenuously for the purpose of securing the attainment of this end; and there can be no question but that the foremost place among those so qualified should be at once taken and held by members of the medical profession. Education is a branch of applied physiology; and the control of the nourishment of the intellectual faculties should be as distinctly a part of medicine as the control of the nourishment of the physical powers. Up to the present time, the former, at least, has been left wholly in the hands of empirics, using the word in its proper and literal sense, and the mental diet provided for the children, even of the rich, has been very much upon a par with the bodily diet provided for the children of the poor; often unwholesome in kind, and erring in the direction of quantity sometimes by deficiency and sometimes by excess. We once made a casual visit to a *crèche*, established by some benevolent ladies as a place in which poor mothers might leave their infants in safe custody during working hours, and at which they were also required to leave a supply of food for the day. The provision thus made for one of the nurslings, a baby of two months' old, consisted of a piece of cheese-rind and a bone of bacon; and it was a very type and pattern of a good deal of what constitutes "schooling," and passes, too often, under the name of education.

If anything can be more grotesque than our system of schooling itself, or better calculated to defeat the objects which it is professedly constructed to attain, it would be the choice of the persons who are appointed to superintend its application. There can be no more curious reading than is furnished by the addresses of the candidates who seek election upon a School Board—that is to say, if we contrast the real duties of such a body with the protestations of those who desire to become members of it. The business of a School Board should be to give such a training as may enable the scholars to think independently about questions which interest either themselves or the class to which they belong, and to arrive at sound conclusions on those questions by the aid of accurate knowledge and well-considered judgment. The ordinary plea of the candidate is his desire to render the exercise of judgment by the scholars superfluous, and to force down their throats some conclusion at which he has arrived himself; and for this object he seeks the assistance of his co-sectaries, religious or political, as the case may be. The characteristic of a free and educated community would be the constant increase of knowledge, the constant diminution of prejudice, the constant enlargement of view—the advancement, in short, of all those changes which the civilisation of the last two or three centuries has wrought among mankind, on the whole, to their great and unquestionable advantage. The tendency of the average candidate is towards some form of stereotype, and he usually seeks to be an educational authority not for the purpose of promoting education, but for the purpose of retarding it so far as inquiry into the value of his own crotchets may be concerned. To such forms of mental infirmity the members of the medical profession are, we think, decidedly less exposed than many other classes of the community; and membership of the educational authorities of the future will afford them positions in which they may render incalculable service to the public. They will know, at least, not only that the *corpus sanum* is of fully equal importance with the *mens sana*, but also that the possession of the former is a condition precedent to the attainment of the latter, and should therefore receive at least equal consideration from any educational authority worthy of the name. They will be likely to compel attention to the prevalence of defects of sense which interfere alike with the reception and with the application of teaching, such as the sub-normal vision which has been found to prevail so extensively among school children, or the imperfections of hearing which the recent researches of Mr. Cheatle compel us to regard as being in all probability equally widely diffused. That gentleman, among 1,000 children between the ages of 3 and 16, in the Hanwell District Schools, found only 423 in whom the ears were normal, and 520 in whom the hearing was more or less deficient. It was possibly one of the latter who, after careful listening to an elementary lesson in geography, replied to a question by saying that the equator was "a menagerie lion running round the earth."

